# The Emerging Circadian Phenotype of Borderline Personality Disorder: Mechanisms, Opportunities and Future Directions

**DOI:** 10.1007/s11920-021-01236-w

**Published:** 2021-04-09

**Authors:** Niall M. McGowan, Kate E. A. Saunders

**Affiliations:** 1 Department of Psychiatry, University of Oxford, Warneford Hospital, Oxford, OX3 7JX UK; 2 Oxford Health NHS Foundation Trust, Warneford Hospital, Oxford, UK; 3 NIHR Oxford Health Biomedical Research Centre, Oxford, UK

**Keywords:** Circadian, Personality disorder, Actigraphy, Melatonin, Chronotype

## Abstract

**Purpose of Review:**

We review the recent evidence suggesting that circadian rhythm disturbance is a common unaddressed feature of borderline personality disorder (BPD); amelioration of which may confer substantial clinical benefit. We assess chronobiological BPD studies from a mechanistic and translational perspective and highlight opportunities for the future development of this hypothesis.

**Recent Findings:**

The emerging circadian phenotype of BPD is characterised by a preponderance of comorbid circadian rhythm sleep-wake disorders, phase delayed and misaligned rest-activity patterns and attenuated amplitudes of usually well-characterised circadian rhythms. Such disturbances may exacerbate symptom severity, and specific maladaptive personality dimensions may produce a liability towards extremes in chronotype. Pilot studies suggest intervention may be beneficial, but development is limited.

**Summary:**

Endogenous and exogenous circadian rhythm disturbances appear to be common in BPD. The interface between psychiatry and chronobiology has led previously to novel efficacious strategies for the treatment of psychiatric disorders. We believe that better characterisation of the circadian phenotype in BPD will lead to a directed biological target for treatment in a condition where there is a regrettable paucity of accessible therapies.

**Supplementary Information:**

The online version contains supplementary material available at 10.1007/s11920-021-01236-w.

## Introduction

Borderline personality disorder (BPD) is a psychiatric disorder characterised by persistent mood instability, impulsivity, identity disturbance and interpersonal dysfunction. Among clinical populations BPD is the most commonly diagnosed personality disorder affecting 10% of psychiatric outpatients and between 15-25% of inpatients [1]. Functional impairment is substantial, involving turbulent interpersonal relationships, poor psychosocial and occupational outcomes [2, 3] and reckless and potentially dangerous behaviours (e.g. substance abuse) [4]. Recurrent self-harm and suicidality are core symptoms, with up to 10% of individuals with BPD dying by suicide [4]. Treatments for BPD are limited. There are no medications specifically licenced for its treatment [5]. Long-term psychotherapies are efficacious [6], but not consistently available [7]. The biological phenotype of BPD has been largely neglected, hindering the innovation of novel translational interventions.

Sleep disturbance and insomnia are common in BPD [8••] but are under-appreciated compared to mood disorders where they form part of the diagnostic criteria. Consequently, the specific sleep complaints of people with BPD are often overlooked and thus untreated [9]. An important process governing sleep that remains under-investigated in BPD is that of the circadian clock. Circadian rhythms are 24-h physiological oscillations that sustain a temporal architecture of sleep/wake behaviour [10]. Circadian rhythm disturbances in behavioural and endocrine measures are implicated in the pathophysiology of several psychiatric disorders and are appreciated clinically as a driver of sleep disturbance [11–13]. Moreover, dimensional traits that also constitute core symptoms of BPD, such as mood instability and impulsivity, are associated with disturbance of circadian rest-activity patterns [14, 15]. A role for altered circadian rhythm function has been proposed in BPD [16•], but to date, this hypothesis has not been elaborated further.

In this review, we apply a chronobiological perspective to the extant BPD sleep literature and review recent studies that specifically examine circadian rhythm function. First, we provide a brief primer on the circadian timing system for a general psychiatry audience. We examine the literature supporting the presence of physiologic circadian rhythm abnormalities in BPD, which consists of studies addressing comorbid circadian rhythm sleep-wake disorders, actigraphy monitoring, heart-rate variability and endocrine measures and a small evidence base supporting chronotherapy for BPD. Given recent calls to articulate borderline pathophysiology dimensionally, we also discuss the association between chronotype and personality traits and the implication this raises for future BPD studies. Finally, we offer recommendations for future research development and clinical practice.

## The Circadian System

### Circadian Entrainment and Circadian Rhythm Sleep Wake Disorders

**Fig. 1:**
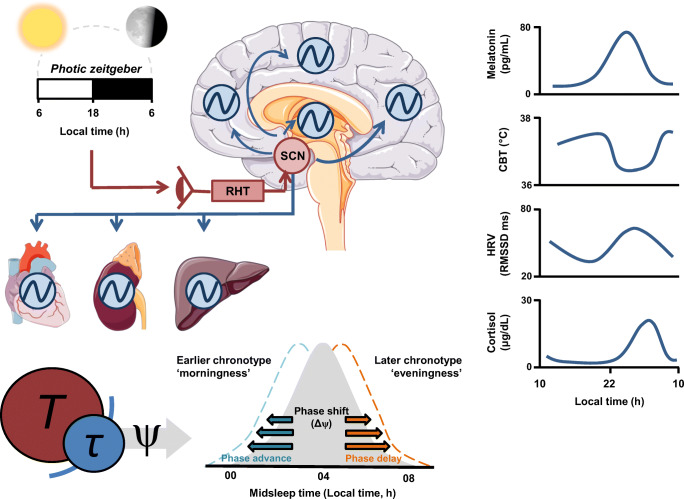
Schematic of the circadian timekeeping system. The circadian time-keeping system is governed by the central clock of the SCN. Efferent SCN signals (blue) coordinate time-keeping among other circadian oscillators in the brain and peripheral organs, establishing a stable phase relation between endogenous circadian oscillators. This network generates an internally stable 24 h cycle that represents the internal day of the circadian system. Circadian signals used to determine the phase of the circadian clock involve robustly oscillating rhythms in core body temperature (CBT), inter-beat interval heart-rate variability (HRV) and plasma melatonin and cortisol. To impart a functional advantage of anticipating time related changes in the environment, the circadian clock is entrained to the external light-dark cycle which serves as the primary zeitgeber to the circadian clock. Retinal photic zeitgeber input signals (red) are transduced to the SCN via a dedicated non-visual circuit known as the retinohypothalamic tract (RHT). Hence, through entrainment, the SCN synchronises its period to that of the 24 h day. Interaction between the 24 h period of the external day (T) and that of the endogenous internal day (tau) generates the circadian phase of entrainment (*ψ*). The phase range at which the internal day coalesces with the external 24 h day gives rise to different ψ expressed as earlier or later chronotypes. Chronotype assessed via the midsleep time displays an approximately normal distribution in the population and is a trait that influences behavioural routines of rest and activity. Manipulation of zeitgeber exposure can produce phase shifts in *ψ*. Early morning exposure to bright light produces a phase advance of the circadian clock, whereas evening exposure produces a phase delay of the circadian clock. Using agents that adjust the phase of the circadian clock is the basis of chronotherapy. Endocrine and temperature reference ranges shown in figure are adapted from Hickie et al. [17] or based on laboratory estimates. RMSSD ranges are based on ambulatory ECG recordings

A schematic overview of circadian clock function is presented in Fig. 1 and a glossary of circadian terminology from this section (italicised terms in text) is presented in the ‘Supplementary Information’ section. The circadian system comprises a network of endogenously oscillating 24-h rhythms known as circadian rhythms that operate across multiple physiological and behavioural domains [18]. This system consists of multiple autonomous and self-sustaining oscillators throughout the brain and periphery. Hierarchical control over the circadian network is exerted by the central circadian pacemaker or ‘master clock’ in the *suprachiasmatic nuclei* (SCN) of the anterior hypothalamus [19]. The master clock aligns the phase of peripheral oscillators to a stable phase throughout the body, establishing a coordinated *internal day* of the circadian clock. Phase and amplitude are primary characteristics of the circadian rhythm. Phase measures the timing of the peak (or trough) of a circadian rhythm relative to a fixed event and is used to situate the rhythm within local time (or relative to sunrise, bedtime, etc.). *Amplitude* measures the difference between peak and trough, and thus quantifies the strength of the circadian output signal.

*Entrainment* is the process by which the circadian system synchronises its internal day relative to that of the environment [20]. Environmental agents that synchronise the circadian system are called *zeitgebers*. In absence of a *zeitgeber* signal, the internal day of the circadian clock oscillates with an intrinsic period that is close to, but deviates slightly from, 24-h; a condition known as *free-running*. Thus, in order to confer a functional advantage of anticipating environmental change, the circadian system establishes a stable phase relation or ‘*phase of entrainment*’ with the environment. Light is the predominant zeitgeber of the human circadian clock [21]. Several qualities of the light/dark cycle determine the expression of circadian rhythms such as the intensity, duration of exposure and time of day of exposure. The output circadian parameter signal may undergo modification of its amplitude (suppression/enhancement) and/or shift its phase (resulting in *phase delay* or *phase advance*).

According to the two-process model of sleep regulation, circadian rhythms interact with homeostatic factors to influence sleep onset time and consolidate a continuous sleep episode [10]. Sleep pressure (homeostatic process) accumulates during wake but is counterbalanced during the day by the circadian process, which exerts an opposing wake-promoting signal. Due to the circadian drive being rhythmic, this wake-promoting signal diminishes in the late evening, a time when homeostatic drive is also high, and in doing so facilitates sleep. Sleep promoting circadian signals involve rhythmic oscillations in core body temperature (CBT), autonomic nervous system (ANS) activity and endocrine rhythms (e.g. cortisol and melatonin) [22, 23], all of which are parameters that exhibit robust circadian rhythmicity even when free-running.

Recurrent abnormal phase relations between the circadian clock and the light/dark cycle produce a misalignment between sleep times and societal and physical 24-h schedules, extremes of which give rise to *Circadian Rhythm Sleep Wake Disorders* (CRSWDs) [24]. Advanced Sleep Wake Phase Disorder (ASWPD) and Delayed Sleep Wake Phase Disorder (DSWPD) refer to abnormally early and late timing of the sleep-wake interval relative to the socially desirable pattern, respectively. Individuals with DSWPD present with chronic sleep-onset insomnia and difficulty waking in the morning, resulting in excessive day-time sleepiness and educational/occupational impairment [25]. Conversely, individuals with ASWPD experience sleepiness in the late afternoon/early evening interfering with social activities, and may experience early-morning awakening insomnia or sleep-maintenance insomnia [25]. Non-24-h sleep-wake disorder (N24SWD) is a condition where a person is unable to entrain their circadian clock to the 24-h light/dark cycle; instead, the clock runs at its intrinsic period, which is typically longer than 24-h [26]. This results in a circadian pattern of sleep/wake scheduling that progressively and perpetually delays each day. N24SWD usually emerges through compromised retinohypothalamic signalling to the SCN and is common in blind people, but assumed very rare in sighted individuals [26, 27].

### Assessment of Circadian Rhythms

The activity of the circadian clock of the SCN cannot be observed directly in humans but its phase and amplitude can be assessed through strongly endogenous circadian rhythms such as are present in CBT and secretion of melatonin and cortisol [28]. Controlled laboratory studies involving constant routine or forced desynchrony protocols are used to observe circadian rhythms in absence of sleep and environmental masking, but are not common in psychiatric populations. Moreover, in situ monitoring is desirable in order to capture circadian processes that pertain to the ecological experience of psychiatric disorder. Several non-invasive and ambulatory methods facilitate assessment in a normal day-to-day environment. Dim-light melatonin onset (DLMO) is the gold standard metric for determining the phase of the circadian clock and can be derived from saliva samples obtained from home at regular intervals under dim-light conditions [29]. Physiologic circadian rhythms can also be observed in cortisol secretion, and passive ambulatory methods such as distal/proximal temperature rhythms, and ANS activity assessed via 24-h ECG [28, 30, 31]. Furthermore, these measures can also be used to reflect the amplitude of the circadian rhythm, which melatonin profiles derived using the DLMO method do not capture.

Circadian rest-activity patterns can be monitored using actigraphy, which is a method widely used in psychiatry research where continuous monitoring of other circadian biomarkers may be prohibitive. A clear advantage of this method is assessment over multiple days, allowing longitudinal monitoring and parallel estimation of sleep [32]. The circadian phase of entrainment can be estimated using actigraph assessed acrophase, midsleep time or non-parametric circadian rhythm parameters [32, 33]. However, these methods are also susceptible to masking effects where activity that is used to infer properties of the underlying circadian rhythm is partially confounded by non-circadian phenomena (e.g. socially enforced schedules, abnormally high or low activity levels, device removal). Despite this caveat, actigraphy provides a useful objective estimate of circadian rhythm function where laboratory routines that examine endogenous clock parameters and eliminate masking effects are unavailable. Study design factors such as appropriate sampling and the use of simultaneous sleep dairies and device removal logs can improve the quality of actigraphy data.

Perhaps the most straightforward instantiation of the circadian phenotype is the emergence of circadian typologies or chronotypes, a trait reflecting either diurnal preference or self-selected sleep scheduling that is used to estimate the time of entrained circadian phase [20, 34]. *Chronotype* is a dimensional trait ranging from morningness to eveningness, alternatively measured by early to late midsleep time. Chronotyping of individuals can be achieved quickly via questionnaires that enquire about daily habits and sleep. Instruments such as the Morningness-Eveningness Questionnaire (MEQ) [35] and Munich Chronotype Questionnaire (MCTQ) [36] are both self-reported methods for assessing chronotype that are validated against sleep diary data and objective measures of the circadian phase.

## Circadian Rhythm Disturbance in Borderline Personality Disorder

Several lines of evidence from sleep studies suggest that circadian rhythm disturbance is a likely contributor to sleep disturbance in BPD. The most recent meta-analytic review of sleep findings in BPD identifies 32 studies [8••]. The most commonly employed method of objectively monitoring sleep in these studies was polysomnography (PSG) with 14 studies published since 1980. Overall, these reviewed findings indicate that sleep disturbance in BPD is characterised by longer sleep onset latency, reduced total sleep time and poor sleep efficiency. Self-reported sleep characteristics using questionnaire instruments also highlight sleep onset latency as a key problem [37–39]. Qualitative examination of BPD experiences reveals complaints of unstructured sleep patterns, and difficulty maintaining a stable sleep-wake routine with associated distress and occupational impairment [9]. The character of sleep disturbance is BPD is concordant with that of circadian phase delay (i.e. the nature of insomnia applies to sleep initiation rather than maintenance). Recently, Fitzpatrick et al. elaborated on these findings using the MEQ to assess chronotype [40•]. In addition to describing greater insomnia severity in BPD compared to healthy controls, their study found greater trait eveningness in BPD compared to healthy controls and clinical controls (generalised anxiety disorder). Controlling for depressive symptoms and medication use suggested that chronotype differences in BPD may also depend on symptom severity.

People with BPD may have a higher risk of being diagnosed with a CRSWD. Dagan et al. reported in two studies a higher prevalence of DSWPD among individuals with personality disorder (including borderline) and vice versa [41, 42]. Early clinical reports describing sighted N24SWD cases have implicated borderline and schizoid psychopathology with the disorder [43, 44]. Reciprocally, individuals with DSWPD and N24SWD exhibit personality traits concordant with borderline personality features [45, 46]. We are unaware of any reported association between ASWPD and BPD. Mechanistic understanding of how CRSWDs might relate to personality disorders is not yet elucidated. Poor entrainment to social zeitgebers, irregular work schedules or unemployment might contribute to DSWPD risk in BPD. For example, shift-work and unemployment may impact the clock by producing a recurring state of circadian misalignment and insufficient/mistimed zeitgeber exposure. Similarly, while the character and pathogenic mechanisms of N24SWD in sighted individuals remain poorly understood, social withdrawal and personality disorder are hypothesised pre-morbid factors [47, 48]. Accordingly, a disrupted sleep phenotype in BPD that is consistent with CRSWD may be overlooked by clinicians, considered secondary to personality disorder psychopathology and social/functional impairment.

Cross-sectional studies directly monitoring circadian rhythms in BPD are limited in number. Verkes et al. were the first to describe abnormal rest-activity rhythms in a sample of majority BPD patients recruited from emergency department admissions [49]. Periodogram analysis of actigraphy data obtained over 5–7 consecutive days revealed a periodicity divergent from 24-h in almost two-thirds of participants. Non-24 h period was associated with worse borderline symptoms, suicidal ideation and disinhibition. Non-24 h activity periods are unusual outside of N24SWD, and in the case of normal day-to-day actigraphy assessment probably reflect between day variability of rest-activity timing rather than an abnormal rhythmic component per se [15]. Indeed, parallel sleep diary results found that improvement in bedtime regularity was associated with symptom improvement [49].

Huynh et al. examined actigraph patterns in an adolescent sample comprised of BPD and bipolar disorder (BD) [50•]. Data were obtained over 9 consecutive days, containing two weekends to capture free-day versus school/work-day differences in behaviour. Overall, BPD participants exhibited more variable night-to-night sleep times compared to healthy controls. On free-days, BPD participants spent more time in bed and had a rise time over an hour later than controls. These findings suggest compensatory oversleeping on free-days consistent with social jetlag; a phenomenon that arises as a function of circadian rhythm misalignment with societal schedules [51]. However, given that the circadian clock is phase delayed during adolescence and social jetlag is more pronounced during school-age, caution is necessary when extrapolating these findings to adults. BPD is also not routinely diagnosed during adolescence.

One of the longest consecutive actigraphy assessment periods recorded in BPD was obtained during the Automated Monitoring of Symptom Severity (AMoSS) study [52]. BPD, BD and healthy control groups were monitored using actigraphy for 28 days. BPD participants exhibited phase delayed rest patterns over 1:30 h later than BD and HC, and phase delayed daytime activity patterns over 2 h later than HC [53••]. Cosinor analysis of distal skin temperature reflected similar delayed patterns in BPD. Furthermore, symptom severity in BPD was strongly associated with circadian rhythm disturbance [54••]. Impulsivity correlated with lower circadian rhythm amplitude and interdaily stability. Mood instability was associated with later activity onset, lower rhythm stability and amplitude, and greater rhythm fragmentation. Notably, mood instability in this study was derived from prospectively reported mood ratings collected for up to 12 months after initial actigraphy recording. Thus, unresolved circadian disturbance may be a predictor of enduring symptom severity. Due to the observational nature of these data, experimental studies are necessary to determine a causal relation between sleep/circadian disturbance and worsened symptom severity in BPD.

An earlier report on a sub-sample of AMoSS participants examined circadian patterns of heart-rate (HR) and activity over a 4 day assessment period using torso mounted holter-like devices containing accelerometer sensors [55•]. Cosinor acrophase of HR, total motor acceleration and vertical acceleration (estimating sleep) revealed desynchrony between HR and activity/sleep in BPD that was greater than BD and absent in controls. These findings suggest internal desynchrony between circadian oscillators in BPD, over and above BD. Moreover, in BPD, mood instability was associated with daily acrophase variability in HR, activity and sleep [56]. Limitations of this approach involve the sampling frequency; inter-beat intervals could not be assessed, and thus, standard time and frequency domain measures of heart-rate variability (HRV) could not be ascertained. Previous studies have examined HRV via ECG in BPD as a measure of ANS activity, but these have focused on short-term resting state recordings [57]. We are aware of only one study that examined 24-h ambulatory ECG in BPD [58]. However, to date, no studies have quantified the circadian rhythm of HRV in BPD.

A number of studies in BPD have examined endocrine parameters, which suggest circadian rhythm abnormality. Rausch et al. and Lieb et al. describe elevated salivary cortisol awakening response (CAR) in female BPD patients [59, 60]. The latter study also demonstrated diurnal elevation until the evening. Wingenfeld et al. report elevated overnight urinary free cortisol in BPD compared to controls [61]. Jogems-Kosterman et al. describe lower morning-to-evening salivary cortisol ratio in BPD compared to controls [62]. These findings suggest greater amplitude of cortisol secretion in response to awakening, and an attenuated 24-h rhythm in the context of elevated diurnal and nocturnal secretion. However, CAR and 24-h cortisol levels are not unambiguous measures of circadian phase or amplitude. The underlying circadian rhythm of cortisol is masked by an ultradian pulsatile secretion pattern that is likely activated further in individuals with alterations in hypothalamus–pituitary–adrenal axis function, as in BPD. The aforementioned cortisol pattern findings may be gender specific (female) and importantly have been shown in medication-free volunteers and controlling for comorbid depression and trauma [59•].

We are aware of only one study that examined melatonin circadian phase in BPD. Bromundt et al. examined melatonin secretion via consecutive saliva sampling during waking hours in a small sample of women (14 BPD compared with 10 healthy controls) [63••]. Melatonin onset time and the phase angle between melatonin onset and bedtime did not differ between groups. Non-parametric indicators of circadian rest-activity function assessed through parallel 3-week actigraph monitoring also showed no differences. As far as we know, no studies have used DLMO as a circadian phase marker in BPD.

Two studies have examined bright light therapy (BLT) in BPD, a treatment modality targeting circadian rhythm function. The aforementioned Bromundt et al. study used a 3-week cross-over design treatment of morning 8000 lx for 30–40 min daily [63••]. BLT led to earlier rise-time, shorter sleep duration and increased nocturnal proximal skin temperature (a circadian measure of vasodilation indexing preparedness for sleep). Daytime alertness and symptoms of atypical depression also improved with treatment. Prasko et al. examined 13 treatment resistant BPD patients with comorbid depression using BLT to augment antidepressant treatment [64•]. The treatment consisted of 6-week application of 1-h morning 10,000 lx BLT added to SSRI treatment. Symptoms of depression and anxiety improved with treatment. However, no measures of sleep or circadian function were employed. To date, there has been no randomised controlled trial of BLT or any other chronotherapeutic or sleep intervention in BPD.

## Dimensional Models of Borderline Personality, Symptoms and Chronotype

The diagnosis of BPD is a categorical one made if five or more of nine behavioural criteria are present [65]. This variety of potential combinations contributes to variability in symptom aetiology, clinical course, and treatment response. That a categorical system may not be optimal for researching or diagnosing personality disorder is a long-standing issue in psychiatry [66]. Personality pathology can also be characterised dimensionally on a continuum, and the majority of the individual diagnostic criteria have been shown to be continuously distributed [67]. The move towards dimensional over categorical approaches to personality disorder classification is reflected in the hybrid dimensional-categorical model included for further study in DSM-5 and the dimensional approach taken in ICD-11. These developments have implications for how circadian rhythm differences may be expressed in BPD and their meaning with respect to symptom severity and management. Evidence from personality studies in the field of chronobiology suggest an association between circadian clock function and traits that predominate in BPD.

Normative personality traits reflected in the five-factor model of personality (FFM) are among the most widely validated psychological phenotypes [68] and have been suggested as analogous dimensions in the study of maladaptive personality traits [69]. Meta-analytic studies of FFM traits in personality disorders highlight that BPD is characterised by high neuroticism, low agreeableness and low conscientiousness, resembling closely core symptoms of mood instability, interpersonal dysfunction and impulsivity, respectively [70, 71]. The overlap between these associations with delayed phase of entrainment suggested by chronotype studies is substantial. Two meta-analytic reviews conclude broadly similar findings. Later chronotype/eveningness is associated with low conscientiousness and low agreeableness [72, 73•]. Furthermore, when morningness is considered a separate dimension on the MEQ (reflecting advanced phase of entrainment), a negative association with neuroticism emerges [73•].

Cloninger’s Temperament and Character Inventory (TCI) [74] is an alternative personality model also commonly used in both BPD and chronobiology research. The TCI assesses four temperament dimensions (novelty seeking, harm avoidance, reward dependence and persistence) and three character dimensions (self-directedness, cooperativeness and self-transcendence). In BPD, temperament dimensions emphasise increased harm avoidance, increased novelty seeking and decreased reward dependence, and character dimensions emphasise decreased self-directedness and decreased cooperativeness, reflecting the clinical impression of the disorder [75, 76]. Chronotype studies indicate that eveningness is positively associated with novelty seeking with mixed findings for harm avoidance [77–81]. Furthermore, character findings suggest that eveningness is negatively associated with self-directedness, cooperation and persistence [77, 79–81].

In healthy populations, symptoms of BPD such as mood instability and impulsivity are both associated with circadian rest-activity pattern disturbance [14, 15]. Eveningness/later chronotype is also a predictor of core BPD symptoms such as dissociative experiences [82], impulsivity [83], anger [84] and suicidal ideation [85]. Hypothesised mechanisms for these relations involve incompatibility between societal demands and chronotype (i.e. social jetlag) and the resultant detriment to sleep. However, there are no studies investigating chronotype and symptom dimensions in BPD and it is not clear how well findings from healthy participants can be extrapolated to BPD. Future chronotype and multimodal circadian phenotyping studies are needed in BPD with a focus on interrogating its polythetic personality and symptom domains.

## Future Research Opportunities and Clinical Recommendations

The studies reviewed here indicate that circadian rhythm disturbance may be an important unmet treatment need for people with BPD and a factor that likely exacerbates the core symptoms of the disorder. It is our view that there is an unexploited opportunity to characterise and therapeutically address circadian rhythm disturbance in BPD, and that doing so may produce significant benefits for those with the diagnosis. We predict that interventions that stabilise circadian rhythm function in BPD will confer benefit for its treatment, particularly in those that exhibit delayed and disrupted sleep patterns. Studies examining the circadian phenotype in unipolar depression and bipolar disorder have demonstrated that circadian rhythm disturbances are a cardinal feature of the chronic sleep disturbance in both disorders [17, 86]. Accordingly, chronotherapeutic interventions (such as BLT) and therapies designed to stabilise 24-h behavioural patterns (such as interpersonal and social rhythm therapy; IPSRT) have been deployed successfully for the treatment of both [17, 87, 88]. Circadian rhythm treatment represents a future directed biological target for intervention in BPD. In contrast to the prolonged nature of psychotherapeutic approaches, chronotherapeutic treatment response is achievable within weeks, and interventions are scalable and can be modified to be delivered remotely. Adjunctive to recommended psychotherapies, chronotherapeutic treatments may widen the treatment options that are available to people with BPD.

Technological advances over the past decade have resulted in enthusiastic support for digital phenotyping in psychiatry; that is in situ monitoring and momentary quantification of individual-level phenotypes from smartphones and personal digital devices [89]. Behavioural circadian patterns are an additional target for future digital phenotyping studies and may be passively monitored using moment-to-moment digital device signals akin to actigraphy use in the field of chronobiology. Examining the impact of circadian rhythm disturbance and misaligned phase on symptom course, decompensation risk and impending crisis presents a novel opportunity for digital phenotyping studies in BPD.

There is also a yet unexplored opportunity to examine the association between dimensions of maladaptive personality and circadian phenotypes, notably chronotype, as has been richly described in the case of normative personality traits. Alternative DSM-5 Model for Personality Disorder [65] proposes a five factor dimensional maladaptive personality trait system that should be used in future studies. We suggest that future studies use a dimensional framework for understanding the impact of circadian rhythm disturbance upon BPD psychopathology. Based on the presentation of BPD and previous chronotype studies among clinical and general populations, it seems likely that domains such as negative affect and disinhibition are more severely impacted by circadian rhythm disturbance than symptoms involving complex interpersonal interactions. Delineation of susceptible features is necessary to innovate targeted approaches for symptom management.

We propose recommendations for future research of circadian clock function in BPD (Table 1) and highlight some clinical recommendations (Table 2). There is preliminary evidence of association between circadian rhythm disturbance and BPD diagnosis and psychopathology. However, this topic needs greater research attention and the causal direction of this relationship has yet to be determined. Future experimental and longitudinal work is necessary to elucidate the mechanisms involved and to track the evolution of symptom severity. An experimental medicine approach will be useful for both establishing the impact of circadian clock function on core BPD symptoms and assessing the efficacy of chronotherapeutic interventions in the disorder. Appreciation for a role of circadian rhythm disturbance in BPD may enable translational therapies for its treatment as has been demonstrated for bipolar disorder [88], a condition with considerable symptom overlap. Feasibility studies in BPD have demonstrated that BLT may be a promising therapeutic avenue [63, 64] that should be further interrogated in a randomised controlled manner.

**Table 1:** Future research recommendations

• Obtain circadian parameters beyond actigraphy in BPD, focusing explicitly on physiologic markers of circadian phase and especially DLMO	
• Design experimental medicine chronotherapy studies to examine causal hypotheses and provide clinical pilot data for future trials	
• Determine the course of BPD symptomatology and how it relates to circadian rhythm disturbance via longitudinal ecological momentary assessment studies	
• Delineate symptom features and dimensional pathological personality traits that are adversely affected by circadian rhythm disturbance in BPD	
• Sleep studies should at a minimum employ parallel measures of chronotype to capture information about the phase of sleep/wake patterns in BPD

**Table 2:** Clinical recommendations

• Sleep should be routinely assessed in personality disorders and detailed psychiatric interview should be conducted to screen for comorbid CRSWDs	
• Consider sleep disturbance and circadian rhythm misalignment as perpetuating and exacerbating factors of core borderline symptoms, especially in the context of occupational and social impairment	
• Emphasise sleep hygiene and social rhythm stabilisation as strategies supplementary to treatment as usual	

Fundamental questions concerning the character of circadian function in BPD remain unaddressed. Most studies have used actigraphy to assess rest-activity rhythms but assessment of other circadian parameters is required. Endocrine and ANS activity measures are variables of interest in BPD studies and express a circadian rhythm that should be examined explicitly. Chronobiological studies have established the DLMO phase of the circadian clock in other psychiatric disorders but this has not yet been examined in BPD. As the purported gold standard marker of clock function, its characterisation will allow important questions to be clarified involving internal phase angle coordination and internal day representation that may be interrupted in BPD.

Clinical practice points should involve routine assessment of sleep disorder in BPD with a particular focus on CRSWDs given their preponderance and reciprocal nature. Sleep complaints from people with BPD should not be considered secondary to core psychopathology. Treatment of sleep disturbance and abnormal sleep/wake phase should be explicit and above usual symptom management for BPD. Until specific evidence-based chronotherapeutic interventions for BPD become available, sleep hygiene and IPSRT inspired approaches with psychoeducation regarding appropriate zeitgeber exposure and social rhythm regularity should be emphasised.

## Conclusions

There is a growing evidence base suggesting circadian rhythm dysregulation in BPD. Recent findings indicate that rest-activity patterns are phase delayed and possibly desynchronised internally from other oscillating autonomic parameters. Reduced circadian rhythm amplitude and daily stability of rest-activity and endocrine rhythms have also recently been reported and these perturbations appear to be associated with worsened core symptoms of the disorder. Chronotype studies in normative samples show consistent associations between later chronotype and traits associated with borderline psychopathology but this trend requires further examination in those with a confirmed diagnosis of BPD. Taken together, the emerging circadian phenotype of BPD suggests a phase delayed clock comporting with earlier clinical reports that describe a greater prevalence of a delayed sleep-wake phase syndrome among people with the disorder. However, further development of this hypothesis is necessary using unambiguous endogenous measures of the clock function (e.g. DLMO). Better classification of the circadian rhythm phenotype in BPD will permit a full characterisation of the impact on patient impairment and enable the development of targeted interventions. Pilot studies find some initial support for BLT but the feasibility of other chronotherapies is not yet clear.

## Footnote

### Search Strategy and Selection Criteria

We selected references for this article from previous work in the field of chronobiology and via a search of AMED, Embase, MEDLINE/Pubmed, PsycINFO and Google Scholar published up until May 31, 2020, using the search terms ‘borderline personality disorder’ or ‘emotionally unstable personality disorder’ and ‘circadian’, ‘chronotype’, ‘biological clock’, ‘actigraphy’, ‘actimetry’, ‘cortisol’ and ‘melatonin’. From these searches, we identified articles published in English and reviewed the citations within these articles. We determined a final list of references based on their relevance to the theme of this review.

## Supplementary Information

ESM 1 (PDF 195 kb)

## Declarations

### Disclaimer

The views expressed are those of the authors and not necessarily those of the NHS, the NIHR or the Department of Health.
