# Supplementary material for: The Emerging Circadian Phenotype of Borderline Personality Disorder: Mechanisms, Opportunities and Future Directions
Source: Curr Psychiatry Rep. 2021 Apr 9;23(5):30. doi: 10.1007/s11920-021-01236-w (PMC8035096; doi:10.1007/s11920-021-01236-w)
Supplement: Supplementary file 1 — (PDF 195 kb) [file 11920_2021_1236_MOESM1_ESM.pdf]

Supplementary Information

The Emerging Circadian Phenotype of Borderline Personality Disorder: Mechanisms, Opportunities, and Future Directions

Niall M McGowan PhD[1,2]*, Kate EA Saunders DPhil[1,2,3]

1. Department of Psychiatry, University of Oxford, Warneford Hospital, Oxford, United Kingdom

2. Oxford Health NHS Foundation Trust, Warneford Hospital, Oxford, United Kingdom

3. NIHR Oxford Health Biomedical Research Centre, Oxford, United Kingdom

ORCID IDs

Niall M McGowan (0000-0001-8183-8558)

Kate EA Saunders (0000-0003-3448-9927)

*Corresponding author: Niall M McGowan

Address: Department of Psychiatry, University of Oxford, Warneford Hospital, Oxford, OX3 7JX, United Kingdom

Email: niall.mcgowan@psych.ox.ac.uk

Phone: +44 1865 613196

Glossary of terms

Amplitude: the crest magnitude of a rhythmic time-series or peak of a fitted curve representing a circadian rhythm. The circadian amplitude indicates the strength of the observed rhythm.

Chronotype: a trait representing either preferred daily times for organising sleep-wake activities on a 'morningness-eveningness' personality dimension, or alternatively a trait representing the phase of entrainment of an individual, usually estimated using midsleep time. In both cases chronotype is used to estimate the underlying phase of the circadian clock.

Circadian Rhythm Sleep Wake Disorders: sleep disorders characterised by abnormal sleep/wake phase and structure with an assumed aetiological basis of underlying circadian rhythm misalignment. CRSWDs described in main text are Advanced Sleep-Wake Phase Disorder (also/previously called Advanced Sleep Phase Type [DSM-5, ICD-10], Advanced Sleep Phase Disorder, Advanced Sleep Phase Syndrome); Delayed Sleep-Wake Phase Disorder (also/previously called Delayed Sleep Phase Type [DSM-5, ICD-10], Delayed Sleep Phase Disorder, Delayed Sleep Phase Syndrome); Non-24-hour Sleep Wake disorder (also/previously called Non-24-hour Sleep-Wake Type [DSM-5], Free-Running type [ICD-10], Hypernychthemeral Syndrome).

Entrainment: process by which the internal day of circadian timing system is synchronised to environmental time cues (zeitgebers) to produce stable phase and period of the circadian rhythm.

Free-running: a condition in which the internal day of the circadian timing system oscillates at its intrinsic and self-sustained period. In humans the free-running circadian period (τ) is slighter greater than 24 hours. In laboratory studies this can be observed in a constant environment without entraining zeitgeber signals. In naturalistic scenarios this may occur where retinohypothalamic signal transmission to the SCN is compromised.

Internal day: the 24-hour representation of the endogenous circadian rhythm generated by the circadian timing system, comprised of the internal time of the SCN and phase alignment with peripheral circadian oscillators.

Phase advance: phase shift of the circadian rhythm such that the phase of entrainment occurs earlier in the day. Circadian rhythms that are earlier than the population norm are referred to as phase advanced.

Phase delay: phase shift of the circadian rhythm such that the phase of entrainment occurs later in the day. Circadian rhythms that are later than the population norm are referred to as phase delayed.

Phase of entrainment: represents a stable phase at which the internal day is embedded to the 24-hour environment; thus used to describe the timing of the circadian rhythm.

Suprachiasmatic nuclei: a bilateral complex located in the anterior hypothalamus that is the region responsible for the coordinated circadian programme of the circadian timing system.

Zeitgeber: environmental time cues that entrain the circadian clock such as the 24-hour light-dark signal. Other relevant time cues involve feeding times and social zeitgebers.
